# Sulfur Mediated Alleviation of Mn Toxicity in Polish Wheat Relates to Regulating Mn Allocation and Improving Antioxidant System

**DOI:** 10.3389/fpls.2016.01382

**Published:** 2016-09-15

**Authors:** Huajin Sheng, Jian Zeng, Yang Liu, Xiaolu Wang, Yi Wang, Houyang Kang, Xing Fan, Lina Sha, Haiqin Zhang, Yonghong Zhou

**Affiliations:** ^1^Triticeae Research Institute, Sichuan Agricultural UniversityWenjiang, China; ^2^Institute of Natural Resources and Geographic Technology, Sichuan Agricultural UniversityWenjiang, China; ^3^College of Resources, Sichuan Agricultural UniversityWenjiang, China

**Keywords:** Mn toxicity, chlorophyll fluorescence, translocation, subcellular distribution, antioxidant defense system, S metabolism

## Abstract

Sulfur (S) is an essential macronutrient that has been proved to play an important role in regulating plant responses to various biotic and abiotic stresses. The present study was designed to investigate the effect of S status on polish wheat plant response to Mn toxicity. Results showed that Mn stress inhibited plant growth, disturbed photosynthesis and induced oxidative stress. In response to Mn stress, polish wheat plant activated several detoxification mechanisms to counteract Mn toxicity, including enhanced antioxidant defense system, increased Mn distribution in the cell wall and up-regulated genes involved in S assimilation. Moderate S application was found to alleviate Mn toxicity mainly by sequestering excess Mn into vacuoles, inhibiting Mn translocation from roots to shoots, stimulating activities of antioxidant enzymes and enhancing GSH production via up-regulating genes involved in S metabolism. However, application of high level S to Mn-stressed plants did not significantly alleviated Mn toxicity likely due to osmotic stress. In conclusion, moderate S application is beneficial to polish wheat plant against Mn toxicity, S exerts its effects via stimulating the antioxidant defense system and regulating the translocation and subcellular distribution of Mn, in which processes GSH plays an indispensable role.

## Introduction

Manganese (Mn) is an essential structural and catalytic microelement of numerous proteins necessary for normal growth, development and stress tolerance of plants (Marschner and Rimmington, 1988). However, Mn is required in small amounts by the plants and is toxic when present in excess (Pittman, 2005). In recent years, with the rapid industrialization, Mn level in soil has escalated due to the mining practice and widespread use of Mn-containing fertilizers and sewage sludge (Luo et al., 2015). This issue is more serious in south of China where acidic soil occupies a proportion of arable land due to the available Mn concentration in these soil is relatively higher (Ren et al., 2007; Millaleo et al., 2010). High level of Mn accumulation in plant consequently disturbs numerous physiological processes like nutrient assimilation, photosynthesis and respiration (Shi and Zhu, 2008; Sheng et al., 2015). A typical Mn toxicity symptoms seen in many plants includes reduction of plant biomass, stunted growth, chlorosis and necrosis (Khabaz-Saberi et al., 2010). Increasing evidence indicates that excess Mn can disrupt protein structures, inactivate enzymes by binding to thiols and displace other essential metals (El-Jaoual and Cox, 1998; Fernando and Lynch, 2015). In addition, Mn is known to disturb redox homeostasis by stimulating the formation of reactive oxygen species (ROS) through Haber–Weiss reaction (Li et al., 2010), which may expose cells to oxidative stress leading to lipid peroxidation, biological macromolecule deterioration, membrane dismantling, ion leakage, DNA-strand cleavage and finally death of plants (Petrov et al., 2015). To fight against heavy metals stress, plants have possessed complex mechanisms for protecting potential cell injury against tissue dysfunction, which including active eﬄux, sequestration and complexion inside the cells by Cys-rich proteins and antioxidant system comprised of enzymatic and non-enzymatic components (Cobbett, 2000; Hall, 2002; Limón-Pacheco and Gonsebatt, 2009).

Optimizing supply of mineral nutrients may alleviate stress-induced negative effects on plants (Nazar et al., 2012). Among the various macronutrients, adequate sulfur (S) supply endows plants with higher tolerance to various stresses, e.g., pathogens, salinity and heavy metal stress (Fatma et al., 2014; Dixit et al., 2015a), because it is an integral part of many defense compounds, such as glutathione, phytochelatins, glucosinolates, and vitamins (Rausch and Wachter, 2005). For example, S could alleviate pathogens-induced membrane disruption and plays a positive role in mustard seedlings against Cd toxicity by inhibiting the Cd translocation from roots to shoots (Anjum et al., 2008; Kruse et al., 2012). In fact, the protective role of S in alleviating HMs like Cd and As was suggested to be closely associated with its participation in regulating the expression of genes involved in the biosynthesis of GSH and PCs, which are in charge of regulating the oxidative status and chelating metals within vacuoles (Gill and Tuteja, 2011). In this process, S is taken up by roots as sulfate from soil by sulfate transporters. After uptake by roots, sulfate is reduced to S^2-^ and then incorporated into cysteine and methionine through a cascade of enzymatic reactions. Finally, cysteine and methionine are used for biosynthesis of GHS and PCs through the sequential action of γ-Glutamylcysteine synthetase (γ-ECS), glutathione synthetase (GS) and phytochelatin synthase (PCS) (Cobbett, 2000).

Wheat is one of the most economically important food crops worldwide. Its cultivation is greatly affected by several environmental factors. Many studies were carried out to investigate the effects of S on metals uptaken and translocation, little information is available on the effects of S on plant defense systems under heavy metal stress. As an important genetic germplasm resource, polish wheat would provide excellent agronomic trait for wheat breeding. It is critical to evaluate its physiological response to adverse condition. Therefore, the present study was designed to explore whether S will alleviate the toxic effects of Mn, and elucidate the possible mechanisms of S-mediated physiological changes of Mn toxicity in polish wheat. The influence of S on Mn-induced changes of growth, photosynthesis, Mn uptake and translocation, subcellular distribution, tissue lipid content, non-protein thiols (NPT), phytochelatin, production of ROS, antioxidant defense enzymes and non-enzymatic antioxidants and gene expression involved in S metabolism in polish wheat seedlings has been studied.

## Materials and Methods

### Plant Materials and Growth Conditions

The experiment was conducted in a chamber of Triticeae Research Institute, Sichuan Agricultural University (Sichuan, China). Uniform seeds of dwarf polish wheat (*Triticum polonicum* L.) (Gene accession No-AS304) were sterilized with 10% sodium hypochlorite for 15 min, rinsed and germinated on perlite irrigated with 1/2 strength Hoagland nutrient solution. One-week-old uniform-sized seedlings were selected and transplanted into plastic barrels (2.5 L) filled with aerated Hoagland nutrient solution (eight plants per barrel). After 2 weeks, different S condition and Mn stress were created by adding additional Na_2_SO_4_ and MnCl_2_ to nutrient medium. The experimental design consisted of eight treatments (CK; S1, 5 mM S; S2, 10 mM S; S3, 25 mM S; Mn, 3 mM Mn; S1′, 5 mM S + 3 mM Mn; S2′, 10 mM S + 3 mM Mn; S3′, 25 mM S + 3 mM Mn). The experiment was arranged in a randomized, complete block design with three replicates. Plants were grown under the following environmental conditions: day/night cycle of 14 h/10 h, at 25°C/18°C, a relative humidity of 70–85% and a light intensity of 200 μmol photons m^-2^ s^-1^. The nutrient solutions were renewed every 4 days. After 2 weeks of treatment, plants were harvested. Roots were dipped in 10 mM EDTA for 10 min, rinsed thoroughly with distilled water, and then separated plants into roots and shoots. Fresh plant material was frozen in liquid nitrogen and stored at -80°C for determining the biochemical and physiological indexes.

### Determination of Plant Growth and Concentrations of Mn and S

Harvested plants were photographed for assessment of toxicity symptom. For determination of mineral element content, the plants were oven-dried for 30 min at 105°C, then at 80°C until the materials reached a constant weight. The dried tissues were weighed, ground into powder and digested in mixed acid [HNO_3_ + HClO_4_ (3:1 v/v)] (Bai et al., 2015) for determination of concentrations of Mn and S, which were measured by inductively coupled plasma spectrometer (IRIS Intrepid II, Thermo Elemental, USA).

### Determination of Mn Subcellular Distribution

0.5 g fresh samples were homogenized in 10 mL of extraction solution containing 0.25 mM sucrose, 50 mM Tris-HCl (pH 7.5), 1 mM DL-dithioerythritol and 5 mM AsA. The homogenate was sieved through a nylon cloth (100 mm mesh size) and the residue was designated as ‘cell wall fraction’. The resulting filtrate was centrifuged at 15,000 r min^-1^ for 45 min. The resultant pellet and supernatant solution were designated as ‘organelle fraction’ and ‘soluble fraction,’ respectively. All operations were undertaken at 4°C. The cell wall and cell organelle fractions were transferred to 100 mL Erlenmeyer flask with de-ionized water, dried and digested with 5 mL of HNO_3_ (Su et al., 2014). Then Mn concentrations were determined by ICP-AES.

### Determination of Chlorophyll and Photosynthesis

The chlorophyll in leaves (0.5 g) was extracted in 25 ml 95% ethanol for 24 h in the dark and determined spectrophotometrically by reading the absorbance at 665 and 649 nm, respectively, according to the method of Knudson (Knudson et al., 1977). The net photosynthetic rate (Pn), transpiration rate (Tr), stomatal conductance (Cond) and intercellular CO_2_ concentration (Ci) were measured with a portable photosynthesis system (LI-6400, LICOR, Lincoln, NE, USA). All the measurements were conducted under the same condition at a constant flow rate of 500 μmol s^-1^ and 500 μmol m^-2^ s^-1^ photosynthetic photon flux density (PPFD). Keep the ambient CO_2_ concentration at 350 ± 5 cm^3^ m^-3^ and the temperature was approximately 25°C.

### Chlorophyll Fluorescence Visualization

Chlorophyll fluorescence images were obtained using a modulated imaging fluorimeter and ImagingWin software (the Imaging PAM M-Series Chlorophyll Fluorescence System, Heinz-Walz Instruments, Effeltrich, Germany) according to Murchie and Lawson (2013). Plants were dark-adapted for 30 min prior to measurement, and the second fully expanded leaves were chosen and measured. The minimum fluorescence (*F*_0_) was obtained by applying measuring light pulses at low frequency (1 Hz) from a light-emitting diode. The maximum fluorescence (*F*_m_) was measured with a saturating pulse of 6000 μmol m^-2^ s^-1^ for 1 s. The image of the maximum quantum efficiency of PSII photochemistry (*F*_v_/*F*_m_) was calculated as [(*F*_m_ -*F*_0_)/*F*_m_]. The current fluorescence yield (*F*_t_) and the maximum light adapted fluorescence (*F*′_m_) were determined by applying an actinic illumination of 400 μmol m^-2^ s^-1^, then ΦPSII was calculated from the formula: (*F*′_m_ -*F*_t_)/*F*′_m_. The coefficient of non-photochemical quenching (qNP) was calculated as (*F*_m_ -*F*′_m_)/(*F*_m_ -*F*′_0_). The coefficient for photochemical quenching, qP, which represents the fraction of open PSII reaction center, was calculated as (*F*′_m_ -*F*_s_)/(*F*′_m_ -*F*′_0_). Image data acquired in each experiment were normalized to a false color scale ranging from 0.00 (black) to 1.00 (purple).

### Determination of Lipid Peroxidation

The level of lipid peroxidation was determined as malondialdehyde (MDA) based on the method of Heath and Packer (1968) with slight modification. Briefly, fresh samples of 0.3 g were ground in 5 ml of 0.1% trichloroacetic acid (TCA). The homogenate were centrifuged at 12000 × *g* for 20 min and 1 mL of the supernatant was mixed with 4 mL of 20% TCA containing 0.5% (w/v) TBA, and subsequently incubated the mixture at 95°C for 30 min and stopped the reaction by placing in an ice bath immediately. After centrifugation at 10000 × *g* for another 5 min, the absorbance of the supernatant at 532 was measured. After subtracting the non-specific absorbance at 600 nm, the amount of MDA was calculated using the extinction coefficient of 155 mM^-1^ cm^-1^ and expressed as nmol g^-1^ FW.

### Histochemical Detection of ROS, Lipid Peroxidation and Loss of Plasma Membrane Integrity

Superoxide ($O_{2}^{•–}$) and H_2_O_2_ were visualized in wheat leaves based on the method of Wang with little modification (Wang et al., 2011). Briefly, staining the second fully expand fresh leaves with 1% 3, 3-dimethoxybenzidine (DAB) for detection of H_2_O_2_ or 0.25 mM nitroblue tetrazolium chloride (NBT) for detection of $O_{2}^{•–}$ under light at 25°C for 8 h. After incubated in NBT or DAB, leaves were washed with distilled water and then decolorized in boiling 95% ethanol which allowed visualization of blue insoluble formazan (for $O_{2}^{•–}$) or deep brown polymerization product (for H_2_O_2_). Opened the leaves with glycerol and photographed immediately. The fluorescence intensity of ROS level in roots was visualized using a fluorescent dye 2′-7′-dichlorodihydrofluorescein diacetate (H_2_DCFDA) as described by Pei et al. (2000). Roots were stained with 50 μM H_2_DCFDA (prepared in 10 mM Tris-HCl buffer containing 50 mM KCl, pH7.2) at room temperature for 20 min, then rinsing the stained roots with Tris–HCl buffer to remove excess dye and observed under a fluorescent microscope. Histochemical detection of lipid peroxidation was performed with Schiff’s reagent (Yamamoto et al., 2001). The roots were incubated in Schiff’s reagent for 20 min, then the stained roots were rinsed with a solution containing 0.5% (w/v) K_2_S_2_O_5_ (prepared in 0.05 M HCl) until the roots color became light red. Loss of plasma membrane integrity was detected in roots with method described by Wang and Yang (2005). The roots were immersed in 10 mL of Evans blue solutions (0.025%, w/v, in 100 μM CaCl_2_, pH 5.6) for 20 min. All the stained roots were photographed after washing with sufficient distilled water three times.

### Assay of Antioxidative Enzymes

For extraction of antioxidative enzymes, 0.5 g fresh samples were ground in 5 mL of 50 mM Tris-HCl buffer (pH 7.0) containing 1 mM EDTA, 1 mM DTT, 5 mM MgCl_2_, 1 mM ASA and GSH (Knörzer et al., 1996). After centrifuging at 12000 × *g* for 20 min, the supernatant was used for enzyme assays. All operations were carried out at 4°C. SOD activity was assayed by measuring its ability to inhibit the photochemical reduction of nitroblue tetrazolium (NBT) following the method of Beauchamp and Fridovich (1971). One unit of SOD activity was defined as the amount of enzyme that inhibited 50% of NBT photoreduction. Ascorbate peroxidase (APX) activity was determined by monitoring the decrease in absorbance at 290 nm as AsA was oxidized according to Nakano and Asada (1981). Dehydroascorbate reductase (DHAR) was determined by measuring the increase in absorbance at 265 nm due to reduced ascorbate formation as described by Nakano and Asada (1981). Glutathione reductase (GR) activity was measured by following the decrease in the absorbance of NADPH at 340 nm for GSSG-dependent oxidation of NADPH, as described by Foyer and Halliwell (1976).

### Determination of Non-protein Thiols (NPT), GSH, and Phytochelatin

The concentration of non-protein thiols (NPT) was measured with the method described by Devi and Prasad (1998). 0.5 g fresh samples were ground with 5 mL of ice-cold 5% sulfosalicylic acid solution in an ice bath and then centrifuged at 10,000 × *g* for 20 min. The supernatant was used for NPT assay using 5, 5-dithio-2, 2-dinitrobenzoic acid (DTNB) as a reagent. Briefly, the reaction mixture contained 1 mL supernatant, 1.85 mL 0.2 M Tris–HCl (pH 8.2) and 0.15 mL 10 mM DTNB was incubated for 20 min. After incubation, the absorbance was measured at 412 nm. Using an aliquot without DTNB to adjust the spectrophotometer to zero absorbance and GSH was used as a standard. GSH was determined according to Nagalakshmi and Prasad (2001). Total glutathione content (GSH plus GSSG) was determined in the homogenates by spectrophotometry at 412 nm, using yeast-GR, DTNB and NADPH. GSSG was determined by the same method in the presence of 2-vinylpyridine and GSH content was calculated from the difference between the total glutathione content and GSSG content. Phytochelatin was measured by using formula: total thiol (NPT) minus GSH (Bhargava et al., 2005).

### RNA Extraction and Quantitative Real Time PCR

Total RNA was extracted using TRIzol reagent (Invitrogen), following the manufacturer’s instructions. Genomic DNA in total RNA samples was digested with DNase I (Omega) prior to first strand cDNA synthesis using the QPCR cDNA synthesis kit (Bio-Rad). qRT-PCR was performed in 96-well plates with CFX-96^TM^ system (Bio-Rad). Each reaction in a total volume of 10 μL containing 1 μL of 15-fold diluted cDNA, 0.5 μL (10 μM) of each PCR primer (**Table 1**), 0.5 μL of ddH_2_O_2_ and 7.5 μL of iTaq^TM^ universal SYBR^®^ Green Supermix (Bio-Rad). PCR reaction condition was set as follows: denaturation at 95°C for 3 min, followed by 40 cycles of 95°C for 10 s and 60°C for 30 s. Melting curves were recorded after the last cycle by heating from 60°C to 95°C with 0.5°C increments. Expression data were normalized with the expression level of Actin by the ΔΔCt method. Real-time PCR experiments were conducted in three independent biological replicates, each consisting of three technical replicates.

**Table 1:** Primer sequences used in quantitative RT-PCR analysis.

	Sequence (5′–3′)
**TpHAST**	
Forward	GTAAGAAGAACCACCACTGACAT
Reverse	TGCTGCTATGAAGGACTACCA
**TpGSH1**	
Forward	AGAGATGTTGAGACGGAAGG
Reverse	CTGTTCTAACAACTTCGTCGAC
**TpGS**	
Forward	GCTCAACACCATCTCAACATCA
Reverse	GAATCAAGACCTAAGACTTCACCAT
**TpPCS1**	
Forward	CTCCTTTATCGTTCTCCTCTTCCT
Reverse	CAGCCTTGCCTTCTCTACCA
**TpActin**	
Forward	CCGATTGCTTGTTATCTGTT
Reverse	GAGGATGAAGACGAGAGTTT

### Statistical Analysis

All the presented data are the mean values of three independent experiments with three replicates. Statistical analyses were performed by analysis of variance (ANOVA) using SPSS software version 20 (SPSS, Chicago, IL, USA). Differences between treatments were separated by the least significant difference (LSD) test at a 0.05 probability level.

## Results

### Plant Growth

Polish wheat treated with excess Mn exhibited visible toxicity symptoms. As show in **Table 2** Mn treatment significantly decreased plant height, biomass and relative water content but increased root length in comparison with controls. The dry weight of roots and shoots were enhanced to a great extent in S1 and S2 treatment. By contrast, all the growth parameters were significantly inhibited in S3 treatment. Application of S at S1 and S2 level was found to enhance the plant height, biomass and relative water content in plants growing under Mn stress. However, no significant changes were observed in these parameters when plants exposed to S3′ treatment.

**Table 2:** Plant height, root length, relative water content and dry weight per 10 plants (DW) of polish wheat seedlings treated with S, Mn and their combination.

Treatment	Plant height (cm)	Root length (cm)	Root (g DW)	Shoot (g DW)	Water %
CK	39.50 ± 1.80 ab	19.17 ± 1.53 c	0.81 ± 0.08 c	3.66 ± 0.27 bc	86.07 ± 1.64 bc
S1	41.50 ± 1.50 a	20.83 ± 1.26 bc	0.92 ± 0.09 ab	4.07 ± 0.11 a	91.32 ± 1.43 a
S2	40.67 ± 1.04 a	19.67 ± 1.76 c	0.95 ± 0.05 a	3.78 ± 0.22 ab	88.56 ± 1.08 b
S3	35.83 ± 1.76 c	16.17 ± 1.53 d	0.63 ± 0.05 d	3.39 ± 0.14 cd	82.25 ± 0.76 d
Mn	31.67 ± 1.76 d	24.26 ± 1.55 a	0.67 ± 0.04 d	2.93 ± 0.13 e	78.36 ± 1.10 e
S1**’**	37.73 ± 1.26 bc	23.19 ± 1.26 ab	0.83 ± 0.01 bc	3.24 ± 0.15 d	85.55 ± 1.18 c
S2**’**	35.67 ± 1.61 c	22.50 ± 0.87 ab	0.85 ± 0.06 bc	3.31 ± 0.09 d	84.18 ± 0.39 cd
S3**’**	32.18 ± 0.76 d	18.33 ± 1.04 cd	0.53 ± 0.04 e	2.75 ± 0.19 e	74.25 ± 1.36 f

*Values are mean ± SD (*n* = 3). Different letters in the same column indicate significant difference at *P* ≤ 0.05.*

### Chl Content, Gas Exchange, and Chlorophyll Fluorescence

Compared with control, S1 and S2 treatment significantly increased Chl a, Chl b, Pn, Cond and Tr but reduced Ci and Chl a/b. The values of Chl a, Chl b, Pn, Cond and Tr in Mn-treated plants was decreased by 49.34, 49.27, 43.33, 55.32, and 47.15%, respectively, compared with the controls (**Table 3**). On the contrary, *C*i was greatly increased by excess Mn treatment. Applying S1 and S2 to Mn stressed plants significantly ameliorated Mn-induced decline on Chl a, Chl b, Pn, Cond and Tr. However, more pronounced ameliorating effect was observed at S2 level. Under Mn stress, S3 had no significant effect on the parameters referred above.

**Table 3:** Photosynthetic pigments content (Chl a and Chl b), net photosynthetic rate (Pn), stomatal conductance (Cond), intercellular CO_2_ concentration (Ci) and transpiration rate (Tr) in polish wheat seedlings treated with S, Mn and their combination.

Treatment	Chl a (mg g^-1^ FW)	Chl b (mg g^-1^ FW)	Chl a/b	Pn (μmol m^-2^s^-1^)	Cond (mol m^-2^s^-1^)	Ci (μmol mol^-1^)	Tr (mmol m^-2^ s^-1^)
CK	2.29 ± 0.17 b	0.69 ± 0.06 c	3.33 ± 0.06 bc	17.54 ± 1.05 b	0.47 ± 0.06 b	274.85 ± 8.39d	11.94 ± 1.70 b
S1	2.47 ± 0.14 ab	0.80 ± 0.04 b	3.09 ± 0.07 d	19.50 ± 0.65 a	0.48 ± 0.04 ab	253.02 ± 8.21 e	13.95 ± 1.78 a
S2	2.63 ± 0.12 a	0.87 ± 0.04 a	3.02 ± 0.04 d	20.94 ± 0.89 a	0.54 ± 0.05 a	278.36 ± 6.58 d	13.59 ± 0.75 ab
S3	1.99 ± 0.16 c	0.58 ± 0.06 d	3.45 ± 0.07 a	15.49 ± 1.01 c	0.39 ± 0.03 c	321.11 ± 16.48 b	8.96 ± 0.76 c
Mn	1.16 ± 0.02 f	0.35 ± 0.01 f	3.30 ± 0.06 c	9.94 ± 0.37 e	0.21 ± 0.02 e	356.18 ± 5.86 a	6.32 ± 0.58 d
S1**’**	1.51 ± 0.05 e	0.45 ± 0.02 e	3.35 ± 0.10 abc	12.46 ± 1.23 d	0.33 ± 0.01d	333.56 ± 6.64 b	8.88 ± 0.80 c
S2**’**	1.78 ± 0.03 d	0.52 ± 0.03 d	3.42 ± 0.03 ab	15.18 ± 0.58 c	0.41 ± 0.02 bc	301.65 ± 12.93 c	8.67 ± 0.56 c
S3**’**	1.27 ± 0.09 f	0.38 ± 0.01 f	3.36 ± 0.05 abc	10.40 ± 0.88 e	0.19 ± 0.01 e	354.66 ± 6.39 a	7.12 ± 0.91 cd

*Values are mean ± SD (*n* = 3). Different letters in the same column indicate significant difference at *P* ≤ 0.05.*

**FIGURE 1:**
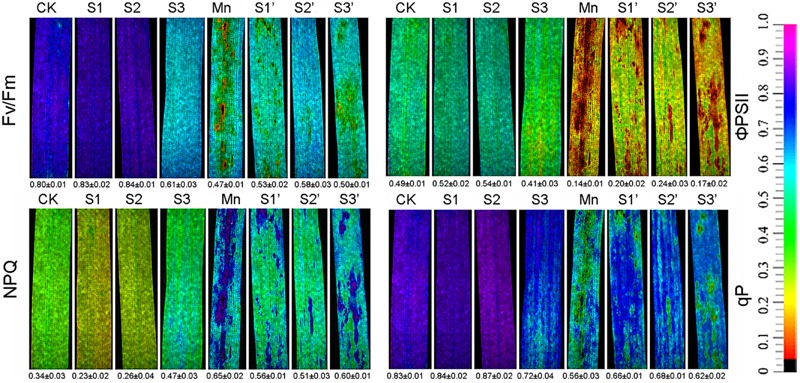
**Chlorophyll fluorescence parameters in polish wheat seedlings treated with S, Mn, and their combination (*F*_v_/*F*_m_, maximum quantum efficiency of photosystem II; qP, photochemical quenching; NPQ, non-photochemical quenching coefficient; and ΦPSII, quantum yield of PSII electron transport)**.

Chlorophyll fluorescence parameter such as *F*_v_/*F*_m_, ΦPSII, qP and NPQ are depicted in **Figure 1**. Results revealed that Mn treatment decreased *F*_v_/*F*_m_, ΦPSII and qP by 41.25, 71.43, and 32.53%, respectively, whereas increased NPQ by 91.18%. S1 and S2 treatment had some stimulations on Fv/Fm, ΦPSII and qP. However, these parameters were suppressed under S3 treatment. S1 and S2 was found to enhance Fv/Fm, ΦPSII and qP but reduce NPQ in Mn-stressed plants, while no obvious changes were observed when the S application reached to S3 level.

### Histochemical Detection of $O_{2}^{•–}$, H_2_O_2_, Lipid Peroxidation and Loss of Plasma Membrane Integrity

**FIGURE 2:**
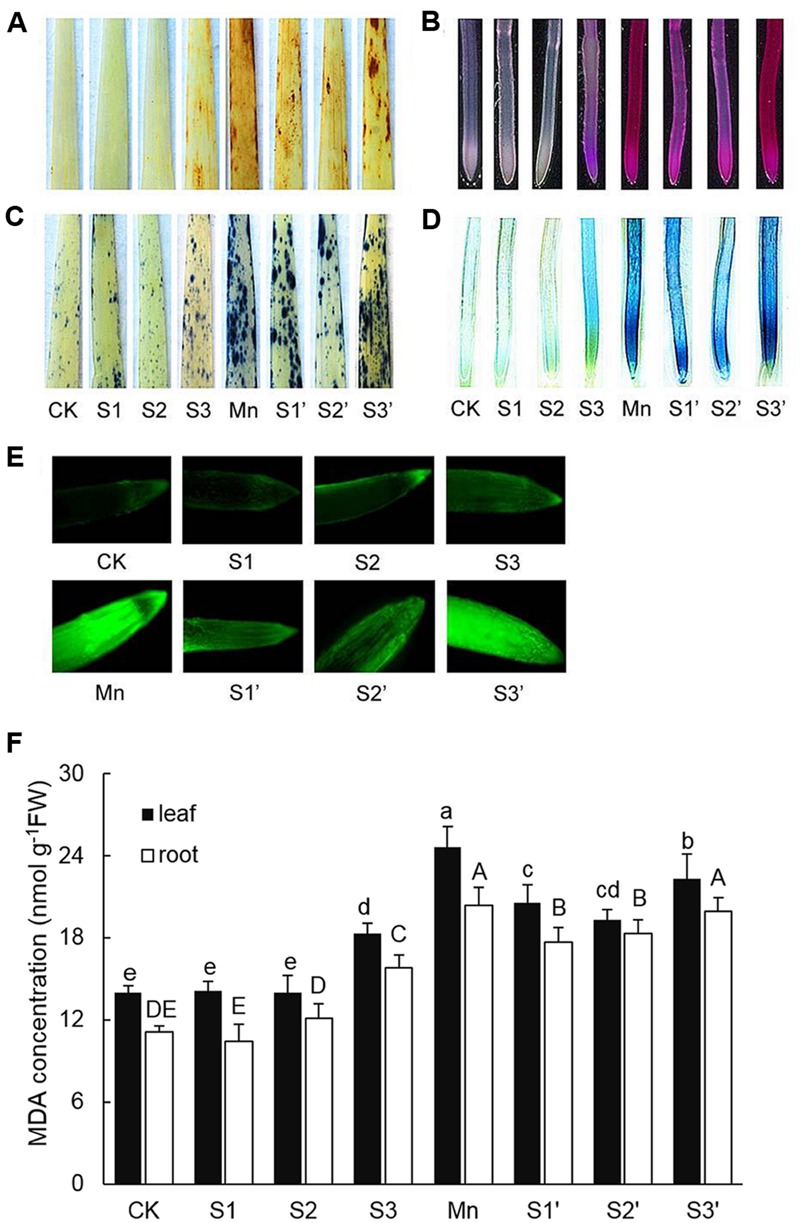
**Histochemical detection of H_2_O_2_ in leaves (A), lipid peroxidation in roots (B), $O_{2}^{•–}$ in leaves (C), loss of plasma membrane integrity in root (D), ROS level in roots (E), MDA contents in leaves and roots (F)**.

The production of $O_{2}^{•–}$ and H_2_O_2_ in leaf were indicated by scattered dark blue spots and brown polymerization products, respectively (**Figures 2A,C**). Compared with control individuals, both $O_{2}^{•–}$ and H_2_O_2_ were up-regulated in leaves of Mn stressed plants. The production of $O_{2}^{•–}$ and H_2_O_2_ did not show any changes under S_1_ and S_2_ treatment, but they were stimulated by S3 treatment. By contrast, under Mn stress condition, S1 and S2 significantly reduced $O_{2}^{•–}$ and H_2_O_2_, with more pronounced reduction observed under S2′ treatment. However, compared with Mn-stressed plants, there is no significant reduction in $O_{2}^{•–}$ and H_2_O_2_ accumulation under S3′ treatment. Mn stress caused significant increases in the degree of peroxidation of membrane lipids and loss of plasma membrane integrity in roots indicated by the heavy staining (**Figures 2B,D**). An obvious increase of these parameters was also detected under S3 treatment. S1 and S2 application was found to alleviate Mn-induced peroxidation of membrane lipids and loss of plasma membrane integrity in roots, with more pronounced effect observed at S2 level. No significant differences in these two parameters were observed between Mn and S3′ treatment. ROS level in roots was visualized by the green fluorescence intensity (**Figure 2E**). Compared with control, exposure of plants to both Mn stress and S3 treatment significantly increased ROS in root. However, the Mn-induced increase in ROS was significantly ameliorated by S1 and S2. S3 had no significant ameliorating effect on ROS in root of Mn-stressed plants.

### MDA Concentration

In response to Mn stress, the concentration of MDA significantly increased in both leaves and roots, which were 1.76 and 1.79 times higher than that of control, respectively (**Figure 2F**). S3 treatment also induced increases of MDA in both tissues of wheat plants. Compared with Mn treatment, application of S to Mn-stressed plants was found to greatly decrease MDA in both tissues except for that in the root of plant under S3′ treatment, where the decrease was not significant.

### Mn and S Accumulation

**FIGURE 3:**
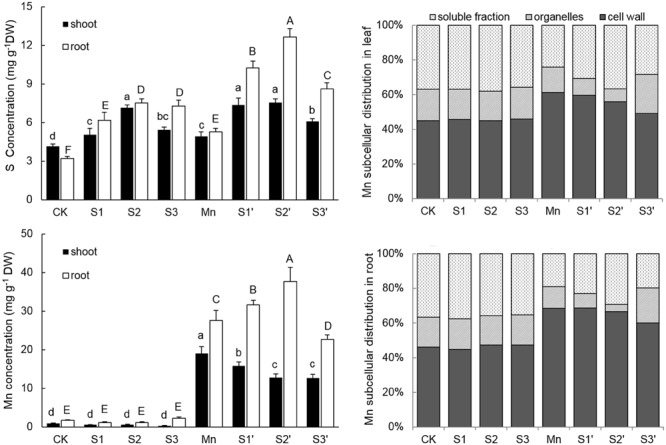
**Mn and S contents and proportion of subcellular distribution of Mn in polish wheat seedlings treated with S, Mn, and their combination.** Data represented as mean ± SD. Different letters on vertical bars indicate significant differences at *P* ≤ 0.05.

Plants treated with Mn showed markedly increased accumulation of Mn in both shoot and root (**Figure 3**). Under no-excess Mn condition, S application had no significant effect on Mn accumulation irrespective of its level, whereas, it significantly influenced Mn contents in Mn stressed plants. Compared with the Mn-treated plants, Mn content was reduced by 16.94 and 32.93% in shoot whereas increased by 14.37 and 36.42% in roots under S1′ and S2′ condition, respectively. However, Mn content in both tissues was significantly decreased under S3′ treatment. Compared with control, Mn treatment increased root S content by 65% and shoot S content by 18.59%, respectively. S application significantly increased S concentrations in both tissues of control and Mn-stressed plants, irrespective of its level. However, S2 proved to be the most effective in enhancing the S concentrations in plants grown under Mn stress, upgrading the values by 53.97% in shoot and 139.77% in root, respectively, when compared with Mn-stressed plants.

### Subcellular Distribution of Mn

The subcellular distribution of Mn in both leaves and roots of wheat plants is shown in **Table 4** and **Figure 3**. Compared with control, Mn treatment increased Mn concentrations in cell wall, organelle and soluble fraction of leaves by about 27, 16 and 12 folds, respectively. Similar results were observed in the roots (**Table 4**). For the proportions of subcellular distribution, Mn treatment significantly reduced the proportions of Mn in the organelle and soluble fraction of both tissues whereas raised that in cell wall by 35.72 and 47.81% for leaf and root, respectively, when compared with the controls (**Figure 3**). In the absence of Mn stress, all the three S treatments had no significant impact on the accumulation and proportion of Mn in different subcellular fractions of both tissues. However, it greatly influenced these parameters in Mn-stressed plants. Compared with Mn treatment, combined excess Mn with both S1 and S2 decreased Mn concentrations in cell wall and organelle of leaves whereas it significantly increased Mn in cell wall and soluble fraction of roots (**Table 4**). Moreover, S1 and S2 were found to enhance the proportions of Mn in soluble fraction while reduce that in organelle for both tissues in Mn-stressed plants, with S2 showed more pronounced effect (**Figure 3**). Under Mn stress, S3 application significantly decreased Mn concentrations in cell wall and soluble fraction of both tissues, but it did not influence Mn content in organelle (**Table 4**). For the proportions of subcellular distribution, compared with Mn treatment, S3′ treatment caused less proportions of Mn accumulated in cell wall but more in organelle (**Figure 3**).

**Table 4:** Subcellular distribution of Mn in leaves and roots of polish wheat seedlings treated with S, Mn, and their combination.

Treatment	Subcellular distribution of Mn in leaves			Subcellular distribution of Mn in roots		
	F_1_ (mg g^-1^ FW)	F_2_ (mg g^-1^ FW)	F_3_ (mg g^-1^ FW)	F_1_ (mg g^-1^ FW)	F_2_ (mg g^-1^ FW)	F_3_ (mg g^-1^ FW)
CK	0.37 ± 0.02 e	0.15 ± 0.01 d	0.31 ± 0.03 c	0.71 ± 0.12 e	0.26 ± 0.01 d	0.56 ± 0.01 ef
S1	0.24 ± 0.01 e	0.09 ± 0.02 d	0.19 ± 0.01 c	0.45 ± 0.03 e	0.18 ± 0.01 d	0.38 ± 0.05 ef
S2	0.24 ± 0.02 e	0.11 ± 0.01 d	0.20 ± 0.02 c	0.47 ± 0.06 e	0.17 ± 0.03 d	0.35 ± 0.03 f
S3	0.12 ± 0.01 e	0.05 ± 0.01 d	0.09 ± 0.02 c	0.93 ± 0.09 e	0.35 ± 0.04 d	0.70 ± 0.06 e
Mn	10.46 ± 1.13 a	2.53 ± 0.44 a	4.11 ± 0.15 a	17.01 ± 1.16 c	3.15 ± 0.14 a	4.71 ± 0.11 c
S1′	8.47 ± 0.59 b	1.39 ± 0.13 b	4.34 ± 0.27 a	19.52 ± 0.80 b	2.39 ± 0.25 b	6.53 ± 0.21 b
S2′	6.42 ± 0.67 c	0.85 ± 0.04 c	4.20 ± 0.20 a	22.56 ± 0.63 a	1.43 ± 0.14 c	9.93 ± 0.38 a
S3′	5.43 ± 0.38 d	2.50 ± 0.25 a	3.13 ± 0.21 b	10.06 ± 0.96 d	3.38 ± 0.11 a	3.30 ± 0.19 d

*F_1_, F_2_, and F_3_ stand for cell wall, organelle and soluble fractions, respectively. Values are mean ± SD (*n* = 3). Different letters in the same column indicate significant difference at *P* ≤ 0.05.*

### Activities of Antioxidant Enzymes

**FIGURE 4:**
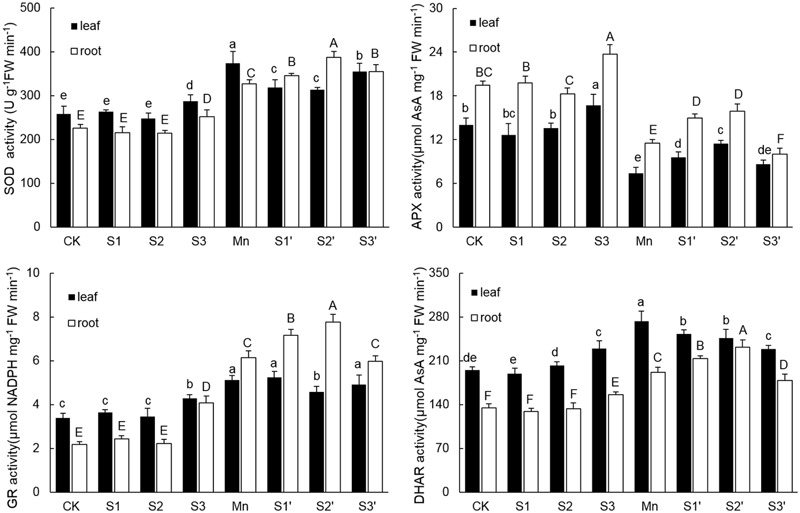
**Activity of SOD, APX, GR, and DHAR in both tissues of polish wheat seedlings treated with S, Mn, and their combination.** Data represented as mean ± SD. Different letters on vertical bars indicate significant differences at *P* ≤ 0.05.

As shown in **Figure 4**. There were no significant differences on activities of SOD, APX, GR and DHAR in both leaves and roots among CK, S1 and S2 treatments. However, the activities of the four antioxidant enzymes in both tissues of plants under S3 treatment were higher than that of controls. Compared with control, Mn stress alone increased activities of SOD, GR and DHAR by 44.85, 51.48, and 39.89% in leaves and 45.27, 182.49, and 42.36% in roots, respectively. In roots of Mn-stressed plants, S1 and S2 application significantly increased the activities of SOD, APX, GR and DHAR, especially for S2. However, except for APX, most of the antioxidant enzymes in leaves of Mn stressed plants were decreased by S1 and S2. S3 application did not change GR activity, but it significantly decreased the activities of SOD in leaf, APX in root and DHAR in both tissues under Mn stress.

### NPT, GSH and Phytochelatin Concentrations

**FIGURE 5:**
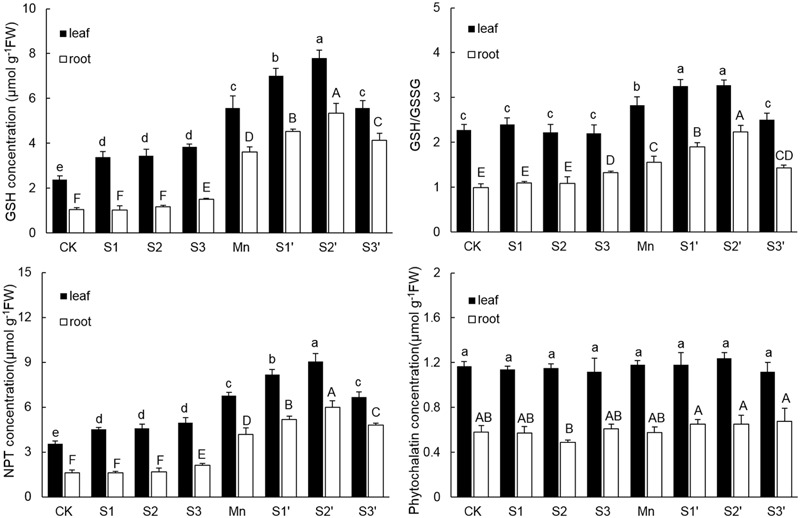
**GSH, NPT, and PCs contents and GSH/GSSG in both tissues of polish wheat seedlings treated with S, Mn, and their combination.** Data represented as mean ± SD. Different letters on vertical bars indicate significant differences at *P* ≤ 0.05.

The components of thiol metabolism were showed in **Figure 5**. Results revealed that the content of GSH in both leaves and roots was significantly enhanced by Mn stress. Without Mn stress, GSH concentration in leaf increased with increasing S application, but no difference existed among the three S levels. Under Mn stress condition, S1 and S2 application resulted in a significant increase of GSH in both tissues, with more pronounced increase observed under S2′ treatment. By contrast, application of S3 to Mn-stressed plants increased GSH only in roots. The change pattern of NPT in both tissues was similar to that of GSH. For GSH/GSSG, regardless of tissue, plants under Mn stress had higher GSH/GSSG than the controls. S3 treatments induced a slight increase of GSH/GSSG in root. However, under Mn stress, GSH/GSSG in both tissues were further up-regulated by S1 and S2. Application of S3 to Mn-stressed plants did not change GSH/GSSG in root, but it significantly decreased that in leaf, which was still slightly increased in comparison to control. Compared with control, Mn stress and S application did not induce a significant change of PCs content in both tissues of polish wheat, except for that in root under S2 treatment. What’s more, regardless of S level and plant tissue, adding S to Mn-stressed plants also had no significant influence on PCs content.

### Expression of Genes Involved in S Metabolism

**FIGURE 6:**
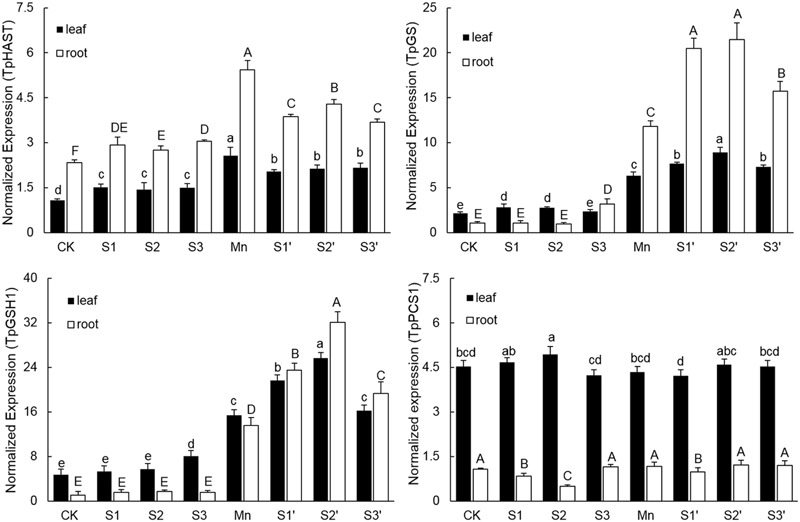
**Expression levels of TpHAST, TpGSH1, TpGS, and TpPCS1 in both tissues of polish wheat seedlings treated with S, Mn, and their combination.** Data represented as mean ± SD. Different letters on vertical bars indicate significant differences at *P* ≤ 0.05.

Without Mn stress, S application slightly up-regulated the transcriptional level of TpHAST (high affinity sulfate transporter) in both leaves and roots. Compared with control, the expression level of TpHAST in Mn-treated plants was up-regulated by 1.43 times in leaf and 1.36 times in root, respectively (**Figure 6**). Regardless of S level and plant tissue, adding S to Mn-stressed plants was found to counteract the Mn-induced up-regulation of TpHAST expression. Mn treatment elevated the abundance of TpGSH1 and TpGS transcripts by 2.29 and 1.93 times in leaf and 11.84 and 10.02 times in root, respectively, when compared with the controls. Both TpGSH1 and TpGS in Mn stressed plants were further increased by S application at all the three levels. The highest expression of TpGSH1 and TpGS were observed under S2′ treatment. Compared with control, Mn stress did not influence the expression of TpPCS1 in both leaves and roots. Except in root of S2′ treatment, S application had no significant influence on the expression of TpPCS1 in both tissues of plants grown under Mn stress (**Figure 6**).

## Discussion

Mn is an essential micronutrient playing an important role in plant growth, but it is toxic when present in excess (Millaleo et al., 2010). Previous studies have showed that excess Mn significantly inhibited plant growth and induced obvious toxicity symptoms (Shi and Zhu, 2008; Arya and Roy, 2011). Our results showed that exposure of polish wheat to Mn stress caused a significant decrease in plant height, biomass and relative water content. The growth inhibition caused by Mn stress might be due to Mn-induced disruption of photosynthesis and nutrient elements balance (Sheng et al., 2015). Out of all the mineral nutrients, S is an important plant nutrient that takes part in plant metabolism and provides vigor to plants under stressful environments (Hardulak et al., 2011). Additional S application could help plants counteract heavy metals (Zhang et al., 2014). In our study, moderate concentration of S (S1 and S2) supply significantly alleviated growth inhibition under Mn stress. The alleviative effects may result from S involved multiple detoxification mechanisms. However, high level S did not significantly alleviate Mn-induced growth inhibition. We inferred this may result from high level S induced osmotic stress which interfering with cellular metabolism and the proper function of S (Zhu, 2001).

Metal-induced inhibition of plant growth has been attributed to their deleterious effects on photosynthetic processes (Dias et al., 2013). In our study, excess Mn significantly inhibited photosynthesis as indicated by the decreased Pn, Cond and Tr rate (**Table 3**). Ashraf and Harris (2013) reported that inhibition of photosynthesis is closely related to the substantial damage to photosynthetic pigments. In consistence with this view, we found excess Mn significantly decreased Chl a and Chl b content in polish wheat plant. In addition, metal caused malfunction of PSII reaction centers might also account for photosynthesis inhibition. Chlorophyll fluorescence is a quick, eco-friendly and non-invasive technique that has been widely used for evaluating fitness of plant under different stress conditions (Singh et al., 2015). In the present study, Mn stress significantly decreased Fv/Fm, ΦPSII and qP whereas increased NPQ values, indicating Mn stress has led to the decline of quantum yield of PSII and increased the need of dissipating excess light energy from PSII. The decline of quantum yield of PSII under heavy metal stress may result from metal caused thylakoid ultrastructure changes and functional photodamage to PSII reaction centers (Ali et al., 2014). In our study, we found moderate concentration of S supply played an effective role in alleviating the photosynthesis inhibition on wheat seedlings subjected to Mn stress. Similar results have been observed in mustard and rice under Cd and As stress, respectively (Anjum et al., 2008; Dixit et al., 2015a). Earlier study reported that wheat cultivars with a higher S assimilation capacity showed high photosynthetic potential under Cd stress (Khan et al., 2007), in our study, we found S application significantly increased S concentrations in both tissues of Mn-stressed plants. Therefore, we suggested that the improvement of photosynthetic capacity with additional S application might partially due to increased demand for S by plants to cope with the negative effects of Mn stress.

Oxidative stress induced by over production of ROS is a common toxic effect shared by different biotic and abiotic stresses. The ROS increase may lead to lipid peroxidation which indicated by MDA level. Therefore, ROS and MDA *in vivo* can indicate the extent of oxidative stress in plants (Shah et al., 2001). In the current study, Mn stress induced a significant damage effect on polish wheat plant indicated by the increased ROS and lipid peroxidation in both tissues of plants. However, moderate S supplementation to Mn stressed plants significantly alleviated Mn induced oxidative stress (**Figures 2** and **3**). The level of ROS in plant cells is strictly regulated by an antioxidant system which comprises enzymatic and non-enzymatic antioxidants. Previous studies demonstrated that stressful conditions could stimulate the antioxidant defense system in different plant species (Zhang et al., 2007). In our study, most of the antioxidants (SOD, GR, DHAR and GSH) showed significant increases in both tissues of plants under Mn stress, which indicates that polish wheat plant has activated antioxidant defense system response to Mn stress. Although most of the antioxidants were upgraded under Mn stress, it was still insufficient to scavenge the excess ROS as indicated by the toxic symptoms. Interestingly, accompanied with the rise of ROS, high level S application also induced significant increases in most of the antioxidants in comparison to control individuals, this may attribute to the fact that high level S application has introduced much Na^+^ to the nutrient solution. The role of S application in alleviating abiotic stresses has proved to be closely related to the efficiently regulated antioxidant defense system (Fatma et al., 2014). In our study, moderate S supplementation to Mn stressed plants further increased all the measured antioxidants (SOD, APX, GR, DHAR, and GSH) in roots. Similarly, Liang et al. (2016) found that application of S to Cd-stressed plants alleviated oxidative stress by promoting the capacity of the AsA-GSH cycle. It is interesting to find that there existed a negative correlation between the oxidative stress and most of the antioxidant enzymes (SOD, GR DHAR) in leaves of Mn-stressed plants supplied with moderate S. This may be associated with S application induced decrease of Mn in shoot that responsible for ROS production. No major stimulation but some inhibition was observed in most of the antioxidant enzymes in Mn-stressed plants (especially in roots) supplied with high level S. In our another study, decreased antioxidant enzyme activity was also found in wheat plants under the combined stress of excess Mn and NaCl due to Na^+^ induced shortage of mineral elements which are essential for the biosynthesis of antioxidant enzymes (not published).

It is generally accepted that keeping toxic metals out of cytosol by sequestering them in cell walls or vacuoles is considered a crucial mechanism for metal tolerance (Su et al., 2014). Previous study showed that Cd-resistant barley accumulated more Cd in the soluble and cell wall fractions than the Cd-sensitive ones (Wu et al., 2005). Cell wall is the first barrier blocking HMs entrance into cells for its negative charge which can bind metal ions and restrict their transportation across the cell membrane (Fu et al., 2011). Weng et al. (2012) reported that most Cd was localized in the cell wall in *K. obovate* under Cd stress. In our study, the distribution of Mn in the cell wall fraction was greatly increased upon Mn treatment, indicating that cell wall deposition was an important defense mechanism against Mn toxicity in polish wheat plant. In the current study, moderate S application to Mn stressed plants was found to increase the proportion of Mn in the soluble fractions while decreased that in organelles, which is in accordance with earlier study showing that S supply promoted Cd transfer to the soluble fraction (Zhang et al., 2014). The major part of metals in the soluble fractions has been demonstrated to be derived from vacuoles which comprise about 90% of the total cell volume (Fu et al., 2011), so we deduced that the protective role of S in alleviating Mn toxicity is partially by sequestering excess Mn in vacuoles. It is well demonstrated that S-mediated Cd or As sequestration in vacuoles was due to S-enhanced synthesis of PCs, which can react with metals forming metal-PCs complex and then sequestered them into vacuoles (Zhang et al., 2014; Dixit et al., 2015b). However, in our study, we did not observed significant difference of PCs among treatments. Actually, PCs synthesis was found to be induced by many HMs, including Cd, Hg, Ag, Cu, Ni, As, and Zn, but there is no evidence that Mn could stimulate PCs synthesis (Hossain et al., 2012). What’s more, Gao et al. (2013) reported that excess Mn treatments did not result in a significant increase in PCs concentrations in both tissues of *Phytolacca americana* L. Because most of the metals sequestered in vacuoles are in forms of metal-thiol complex (Gallego et al., 2012), other ligands may take part in sequestering Mn in vacuoles in present study. Except for PCs, previous studies have showed that GSH can also chelate HMs forming metal-GS complex to facilitate detoxification by vacuolar sequestration (Li et al., 1997). In the current research, we found that moderate S application resulted in higher GSH formation in both tissues of Mn-stressed plants. Thus, the results suggested that S-mediated Mn sequestration in vacuoles of polish wheat plant is more likely through enhancing GSH synthesis to form MnGS complex. Except for keeping toxic metals out of cytosol, many studies in the literature illustrated that HMs sequestration in vacuoles also contributed to limit HMs translocation from roots to shoots (Dixit et al., 2015b; Liang et al., 2016). In agreement with these previous reports, our results showed that adding moderate S to Mn-stressed plants significantly inhibited the translocation of Mn from roots to shoots, and most of the increased Mn in root was distributed in the soluble fractions. Taken together, these results suggested that moderate S could protect polish wheat from Mn toxicity by regulating the translocation and subcellular distribution of Mn via enhancing GSH synthesis. It is interesting to find that adding high level of S to Mn stressed plants caused more proportions of Mn transferred from cell wall to organelle. High level S could introduce much Na^+^ to the solution, so that the decreased Mn accumulated in cell wall by high level S may result from the displacement of Mn by Na^+^ due to its high affinity to the sorption sites on cell wall (Mei et al., 2014). HMs accumulation in the organelle fractions is more detrimental to plant cell than that in cell walls and vacuoles (Hall, 2002). In the current study, although high level S induced pronounced decreases of Mn in both roots and shoots of Mn stressed plants, the concentration of Mn in the organelle did not change much due to the transformation. Therefore, this may be another reason why high level S did not alleviate Mn toxicity.

Sulfur metabolism is a core pathway for the synthesis of molecules like GSH and PCs required for heavy metal tolerance in plants (Hardulak et al., 2011). Up-regulation of various genes involved in S metabolism has been reported in *Crambe abyssinica* under As stress (Paulose et al., 2010). In current study, several important genes involved in S metabolism were selected to study the effect of S application on polish wheat response to Mn stress. Consistent with the concentrations of S and GSH, the expression of sulphate transporter (TpHAST), γ-Glutamylcysteine synthetase (TpGSH1) and glutathione synthetase (TpGS) were significantly up-regulated in both tissues of plants under Mn treatment. These results indicated that more S assimilation and GSH production are needed to overcome Mn toxicity in polish wheat plant. However, compared with Mn treatment, application of S to Mn-stressed plants further up-regulated TpGSH1 and TpGS whereas down-regulated TpHAST in both tissues, Similarly, Dixit et al. (2015b) reported that S supplementation to As exposed plants reduced the expression level of sulfate transporters while up-regulated most of the enzymes involved in S assimilatory pathways and downstream thiolic metabolites. Additional S application met the S demand of Mn-stressed plants may be a reasonable explanation for the down-regulated TpHAST, but the exact mechanisms for S application induced further up-regulation of TpGSH1 and TpGS in Mn-stressed plants remains to be examined in future. Consistent with the concentration of PCs, the expression of TpPCS1 was not affected by Mn exposure or S application, which confirms the fact that PCs did not play an important role in Mn detoxification (Gao et al., 2013).

## Conclusion

Our results showed that Mn stress inhibited the growth of polish wheat plant by interfering with photosynthesis. The toxic effects of Mn mainly resulted from the oxidative stress induced by excess Mn accumulation. In response to Mn stress, polish wheat plant activated antioxidant defense system, accumulated more Mn in the cell wall and enhanced S assimilation to counteract Mn toxicity. Moderate S application would alleviate Mn toxicity by restraining Mn translocation from roots to shoots, sequestering excess Mn into vacuoles likely in the form of GSH-Mn complex, reducing oxidative stress through stimulating activities of antioxidant enzymes and enhancing GSH production by further up-regulating genes involved in S metabolism. In contrast, high S application did not significantly alleviated Mn toxicity, likely due to high level S induced osmotic stress which interfering cellular metabolism. These findings showed that wheat plant adjust their response to excess Mn by S nutrient supplementation, including the elaborated regulation of Mn distribution and ROS elimination.

## Author Contributions

YZ and JZ designed the research. HS, YL, and XW performed the research. HS, YW, HK, XF, LS, and HZ analyzed the data and finally HS and JZ wrote the article.

## Conflict of Interest Statement

The authors declare that the research was conducted in the absence of any commercial or financial relationships that could be construed as a potential conflict of interest.
